# The incidence of low venous oxygen saturation on admission to the intensive care unit: a multi-center observational study in The Netherlands

**DOI:** 10.1186/cc6811

**Published:** 2008-03-04

**Authors:** Paul A van Beest, Jorrit J Hofstra, Marcus J Schultz, E C Boerma, Peter E Spronk, Michael A Kuiper

**Affiliations:** 1 Medical Center Leeuwarden, Leeuwarden, The Netherlands; 2 University Medical Center Groningen, Groningen, The Netherlands; 3 Academic Medical Center, University of Amsterdam, Amsterdam, The Netherlands; 4 HERMES Critical Care Group, The Netherlands; 5 Gelre Hospitals, location Lucas, Apeldoorn, The Netherlands

## Abstract

**Background:**

Low mixed or central venous saturation (S(c)vO_2_) can reveal global tissue hypoxia and therefore can predict poor prognosis in critically ill patients. Early goal directed therapy (EGDT), aiming at an ScvO_2 _≥ 70%, has been shown to be a valuable strategy in patients with sepsis or septic shock and is incorporated in the Surviving Sepsis Campaign guidelines.

**Methods:**

In this prospective observational multi-center study, we determined central venous pressure (CVP), hematocrit, pH, lactate and ScvO_2 _or SvO_2 _in a heterogeneous group of critically ill patients early after admission to the intensive care units (ICUs) in three Dutch hospitals.

**Results:**

Data of 340 acutely admitted critically ill patients were collected. The mean SvO_2 _value was > 65% and the mean ScvO_2 _value was > 70%. With mean CVP of 10.3 ± 5.5 mmHg, lactate plasma levels of 3.6 ± 3.6 and acute physiology, age and chronic health evaluation (APACHE II) scores of 21.5 ± 8.3, the in-hospital mortality of the total heterogeneous population was 32.0%. A subgroup of septic patients (*n *= 125) showed a CVP of 9.8 ± 5.4 mmHg, mean ScvO_2 _values of 74.0 ± 10.2%, where only 1% in this subgroup revealed a ScvO_2 _value < 50%, and lactate plasma levels of 2.7 ± 2.2 mmol/l with APACHE II scores 20.9 ± 7.3. Hospital mortality of this subgroup was 26%.

**Conclusion:**

The incidence of low ScvO_2 _values for acutely admitted critically ill patients is low in Dutch ICUs. This is especially true for patients with sepsis/septic shock.

## Background

Global tissue hypoxia as a result from systemic inflammatory response or circulatory failure is an important indicator of shock preceding multiple organ dysfunction syndrome (MODS). The development of MODS predicts outcome of the septic patient [1]. Unrecognized and untreated global tissue hypoxia increases morbidity and mortality. Accurate detection of global tissue hypoxia is therefore of vital importance. Physical findings, vital signs, measuring central venous pressure (CVP) and urinary output are of the utmost importance, but not always sufficient for accurate detection of global tissue hypoxia [2,3].

It is now generally accepted that a decreased central venous oxygen saturation (ScvO_2_) obtained from a central venous catheter, can reveal a mismatch between oxygen supply and oxygen demand, hence global tissue hypoxia [1]. Decreased values predict poor prognosis after cardiovascular surgery [4], in severe cardiopulmonary disease [5], and in septic or cardiogenic shock [6,7]. ScvO_2 _and SvO_2 _(mixed venous oxygen saturation) can therefore be used as hemodynamic goals during resuscitation. According to Rivers *et al*. [8], hemodynamic optimization demands 'early goal-directed therapy' (EGDT), including ScvO_2_-guided treatment. It was concluded that goal-oriented manipulation of cardiac preload, afterload and contractility, to achieve a balance between systemic oxygen delivery and oxygen demand, is a valuable strategy in patients with sepsis or septic shock during the resuscitation period in the emergency department (ED) [8]. More recently, as a result of this study, an EGDT treatment protocol was included in the 'Surviving Sepsis Campaign' guidelines [1]. Also, several studies on implementation of such a protocol, partially in combination with other recommendations, have been published over the last years [9-11].

Based on clinical experience it seemed that the syndrome targeted in the EGDT study [8] was not very common in intensive care units (ICUs) in The Netherlands, and thus EGDT was not commonly indicated. The main purpose of this study was to determine the incidence of low ScvO_2 _values in our geographical setting. We monitored a heterogeneous group of critically ill patients during unplanned admission in three Dutch multidisciplinary ICUs. Also, illustratively, we compared the subgroup of septic patients with the population of septic patients as described by Rivers *et al*. [8], with respect to ScvO_2 _and other baseline characteristics.

## Methods

### Study centers

We studied ICU populations in one academic ICU (the Academic Medical Center (AMC) in Amsterdam, The Netherlands) and two non-academic ICUs (Gelre Hospital (GH) location Lukas, in Apeldoorn, The Netherlands; Medical Center Leeuwarden (MCL) in Leeuwarden, The Netherlands). The AMC is a large teaching hospital where the ICU is a 28-bed 'closed format' department in which medical/surgical patients, including cardiothoracic and neurosurgical patients, are being treated. GH is an affiliated teaching hospital where the ICU is a 10-bed 'closed format' department. MCL is a large general teaching hospital in the north of The Netherlands, with a 14-bed 'closed format' mixed medical/surgical ICU, including cardiothoracic patients.

### Patients and data collection

Between January 2006 and March 2007 a total of 340 patients, all 18 years or older, with a clinical indication for a central venous catheter (CVC) (BD Medical Systems, Singapore), pulmonary artery catheter (PAC) (Edwards Lifesciences LLC, Irvine, CA, USA) or Continuous Cardiac Output (CCO) catheter that measures SvO_2 _continuously (Arrow Deutschland GmbH, Erding, Germany) were recruited. Indication for a central venous, PAC or CCO catheter was left to the discretion of the attending physician. The patients arrived into the ICU either directly from the ED, from the general ward, or after acute surgery with severe sepsis, septic shock or cardiogenic shock, respiratory failure, central nervous problems, and other acute conditions. In the EDs, there was no standardized protocol for hemodynamic treatment of septic patients. Fluid resuscitation was mostly guided by blood pressure monitoring. Inotropes were given scarcely at the study EDs. Intubation in the ED was also uncommon. In the operating theatres no ScvO_2_/SvO_2 _measurements took place, nor any kind of goal-directed therapy implemented. All patients were treated according to standard practice for the ICU. Exclusion criteria were elective surgery and aged < 18 years. Collection of data for observational study without informed consent was approved by the Medical Ethics Committees of all three hospitals.

Measurements of systolic arterial pressure (SAP), mean arterial pressure (MAP) and central venous pressure (CVP) were recorded immediately after arrival into the ICU. Hematocrit (Hct), lactate plasma levels and pH were determined from the first obtained arterial blood sample in the ICU.

Acute physiology, age and chronic health evaluation (APACHE) II score [12] and sequential organ failure assessment (SOFA) score [13] at the time of admission into the ICU were also collected.

### Statistical analysis

The statistical package SPSS 15.0.1 for Windows (SPSS Inc., Chicago, IL, USA)) was used for statistical analysis. All data were tested for normal distribution with the Kolmogorov-Smirnov test before further statistical analysis. Differences between populations were assessed using the Student's paired t test (normally distributed data). Data were displayed as mean ± SD. Statistical significance was assumed at p < 0.05.

## Results

### Patients

A heterogeneous population with a total of 340 critically ill patients was evaluated in the three participating ICUs (Table 1). The patients arrived into the ICU either directly from the ED (*n *= 135; 40%), from the general ward (*n *= 126; 37%) or after acute surgery (*n *= 79; 23%). To determine ScvO_2 _or SvO_2 _values, central venous or mixed venous oxygen saturation was measured as early as possible after insertion of a central venous catheter (*n *= 263) or pulmonary artery/CCO catheter (*n *= 77). The vast majority (93%) of the patients were enrolled within 6 h after presentation in the ER. More than 99% of all the data was obtained within 2 h after ICU admission. The numbers of measurements of central or mixed oxygen venous saturation were not normally distributed between the three ICUs. In all three hospitals the mean SvO_2 _was higher than 65% and mean ScvO_2 _was higher than 70%. Overall in-hospital mortality of our population was 32.0%.

**Table 1:** Distribution of clinical problems in the three ICUs

Admission diagnosis	MCL (*n *= 93)	GH (*n *= 138)	AMC (*n *= 109)	Total (*n *= 340)
Sepsis/septic shock	47 (51)	64 (46)	39 (36)	150 (44)
Cardiac failure, cardiac arrest	28 (30) 10	36 (26) 10	31 (28) 17	95 (28)
Respiratory failure	7 (8)	13 (10)	12 (11)	32 (9)
CNS	5 (5)	7 (5)	10 (9)	22 (7)
Other	6 (6)	18 (13)	17 (12)	41 (12)

Data are presented as numbers (%). AMC, Amsterdam Medical Center; CNS, central nervous system; GH, Gelre Hospital; ICU, Intensive Care Unit; MCL, Medical Center Leeuwarden.

In 263 patients venous oxygen saturation was measured centrally (Table 2). Mean ScvO_2 _was 72.0 ± 12.3%. A total of 38 patients (14%) had a ScvO_2 _< 60%, and only 14 (5%) patients had a ScvO_2 _< 50%. While only a single patient of the latter group was in septic shock, in-hospital mortality of these 14 patients was 57% (8/14).

**Table 2:** Demographic data, variables and outcome data; comparisons of sepsis patients with EGDT study [8] data

Variable	Present cohort (*n *= 263)	Sepsis (*n *= 125)	EGDT study (*n *= 263)	p Value^a,b^
Age (years)	67.3 ± 14.2	68.9 ± 13.5	65.7 ± 17.2	0.01*
Female (%)	41	38	49.4	
Male (%)	59	62	50.6	
Heart rate (beats/min)	107 ± 27	115 ± 26	115 ± 29	1.0
CVP (mmHg)	9.8 ± 5.4	10.8 ± 4.9	5.7 ± 8.5	< 0.01*
MAP (mmHg)	58 ± 16	60 ± 13	75 ± 25	< 0.01*
ScvO_2 _(%)	72.0 ± 12.3	74.0 ± 10.2	48.9 ± 12.3	< 0.01*
Lactate (mmol/l)	3.3 ± 3.3	2.7 ± 2.2	7.3 ± 4.6	< 0.01*
Arterial pH	7.33 ± 0.12	7.35 ± 0.10	7.32 ± 0.18	0.42
Hematocrit (%)	31.0 ± 7.0	30.3 ± 6.9	34.7 ± 8.5	< 0.01*
APACHE II score	21.5 ± 8.5	20.9 ± 7.3	20.9 ± 7.2	1.0
SOFA score	9.5 ± 3.6	9.6 ± 3.0		
In-hospital mortality (%)	31.0	26.0		
Standard therapy			46.5	
EGDT			30.5	

Data are presented as means ± SD. ^a^Unpaired t test; ^b^sepsis subgroup vs EGDT study. *Statistically significant difference. APACHE II, Acute Physiology, Age and Chronic Health Evaluation; CVP, central venous pressure; EGDT, early goal-directed therapy; MAP, mean arterial pressure; ScvO_2_, central venous oxygen saturation; SOFA, Sequential Organ Failure Assessment.

### Septic patients

In patients with sepsis or septic shock (*n *= 150) central venous oxygen was measured in 125 patients and mixed venous oxygen saturation was measured in 25 patients. The in-hospital mortality of our septic patients was 27%. A total of 73 patients arrived in the ICU from general wards (49%). The mean ScvO_2 _value was normal: 74.0 ± 10.2%. Only eight (6%) patients had a ScvO_2 _< 60%, and one (1%) < 50% (Table 2).

### Comparison with the EGDT population

Compared to the Rivers study group our septic patients revealed a significantly higher ScvO_2 _(74.0 ± 10.2 vs 48.9 ± 12.3%; p < 0.01), lower lactate plasma levels (2.7 ± 2.2 vs 7.3 ± 4.6 mmol/l; p < 0.01), and lower hematocrit (30.3 ± 6.9 vs 34.7 ± 8.5%; p < 0.01). A total of 83% of patients needed endotracheal intubation versus 55% in the EGDT study [8]. APACHE II scores were equal (20.9 ± 7.0 vs 20.9 ± 7.2; p = 1.0). The in-hospital mortality of this subgroup was 26% (Table 2).

### Mixed venous oxygen saturation

Measurement of mixed venous oxygen saturation took place in 77 patients. Mean SvO_2 _was 68.2 ± 11.8%. Mean lactate was 4.3 ± 4.2 mmol/l; arterial pH was 7.30 ± 0.11. With mean APACHE II scores of 21.8 ± 7.3 and mean SOFA scores of 9.3 ± 3.6, the in-hospital mortality was 37% (Table 3).

**Table 3:** Demographic data, variables and outcome data; mixed venous saturations

Variable	Present cohort (*n *= 77)	Sepsis (*n *= 25)
Age (years)	61.7 ± 14.0	65.4 ± 10.4
Female (%)	39	52
Male (%)	61	48
Heart rate (beats/min)	102 ± 21	102 ± 21
CVP (mmHg)	13.0 ± 4.9	13.7 ± 4.6
MAP (mmHg)	61 ± 15	61 ± 13
SvO_2 _(%)	68.2 ± 11.8	72.1 ± 10.8
Lactate (mmol/l)	4.3 ± 4.2	3.3 ± 2.3
Arterial pH	7.30 ± 0.11	7.32 ± 0.08
Hematocrit (%)	29.9 ± 7.1	28.2 ± 5.4
APACHE II score	21.7 ± 7.3	22.2 ± 5.4
SOFA score	9.3 ± 3.6	10.3 ± 3.7
In-hospital mortality (%)	37.0	28.0

Data are presented as means ± SD. APACHE II, Acute Physiology, Age and Chronic Health Evaluation; CVP, central venous pressure; MAP, mean arterial pressure; SOFA, Sequential Organ Failure Assessment; SvO_2_, mixed venous oxygen saturation.

Of the 25 patients in whom mixed venous oxygen saturation was measured in the subgroup of septic patients, four (16%) patients showed a SvO_2 _value < 60% on admission. One patient (4%) had a SvO_2 _< 50%. In this relatively small subgroup, mean Hct was 28.2 ± 5.4%, mean MAP 61.0 ± 13.4 mmHg and mean CVP 13.7 ± 4.6 mmHg, while 80% (20/25 patients) needed mechanical ventilation. Mean APACHE II score was 22.2 ± 5.4 and mean SOFA score10.3 ± 3.7. The in-hospital mortality of this subgroup was 28% (Table 3).

## Discussion

The main result of this present multi-center observational study is the low incidence of low ScvO_2 _values (< 50%) in septic patients being only 1%. Secondary findings were the normal mean ScvO_2 _values and normal mean SvO_2 _values in critically ill patients, including patients with severe sepsis or septic shock, on admission in the three ICUs.

Development of severe sepsis and septic shock involves several pathogenic changes, including global tissue hypoxia as a result of circulatory abnormalities [14]. In particular, hemodynamic optimization as a therapeutic target has been studied over the last decade [2,8,9,15,16]. Based on promising results from earlier studies [2], Rivers *et al*. randomized patients with severe sepsis or septic shock to standard therapy or EGDT. The latter resulted in an absolute reduction in 28-day mortality of 16% [8]. Improvement of the balance between oxygen delivery (DO_2_) and oxygen demand (VO_2_) played an important role. Other studies, however, found no reduction of morbidity or mortality as a result of aggressive hemodynamic optimization, despite higher central venous oxygenation or lower lactate concentrations [15,16]. Studies that enrolled patients admitted into the ICU were unable to show a decrease in mortality rate after aggressive hemodynamic optimization [16,17], in contrast to studies that implemented certain treatment protocols, including antibiotic therapy, in the emergency department [8,9,11]. In this ICU study we found mean ScvO_2 _and SvO_2 _values in the normal range. Similar figures are described previously in the later stage of sepsis and in ICU patients [18,19]. This is in concordance with the findings by Gattinoni *et al*. (67, 3–69, 7%) [15] and Bracht *et al*. (70%) [20].

ScvO_2 _is a surrogate for SvO_2_: a significant correlation between the two has been described [21]. Although it might still be debatable whether central venous and mixed venous oxygen saturation are equivalent or not [18,19,21], the clinical importance of both measurements seems not to be an issue. The Surviving Sepsis Campaign recognizes such in the resuscitation portion of its severe sepsis and septic shock information [1]. Our study design does not allow for any statistical evaluation of ScvO_2 _compared to SvO_2_.

APACHE II scores were similar in comparison to the population described in the EGDT study [8]. This suggests equal mortality rate predictions. However, physiological scores such as APACHE II are dependent on variables that reflect the progression or reversal of organ dysfunction. Treatment in the ED or operating theatre prior to ICU admission influences calculation of the physiological scores. Consequently, the physiological scores at our ICUs could partially be underestimated. The significantly higher lactate plasma levels in the EGDT study suggest a more severe tissue hypoperfusion in that group. However, it is the clearance rate that is associated with less organ failure and improved survival [22].

Unlike significantly lower mean arterial pressures, the higher CVP and the lower hematocrit suggest that the septic patients were less hypovolemic compared to the EGDT population. Relatively high mean blood pressure in the EGDT population suggests an earlier stage of sepsis with predominating vasoconstriction, or pre-existing hypertension. The higher CVP in the subgroup with septic patients (*n *= 125) is partially the result of high percentage of endotracheal intubation and thus increased intrathoracic pressure before measurement (83%). In the EGDT study, less than 55% needed intubation at admission.

As mentioned earlier, in the present study the patients were treated in the ED, or elsewhere, before admission to the ICU. This treatment was different from the treatment given in the EGDT study. Nevertheless, our patients received some fluid therapy. Transfusion of red blood cells in our EDs was based on clinical suspicion or evidence of severe blood loss and not on low hematocrit only. Also, a main principle of treatment is to improve oxygen delivery and this could contribute to higher ScvO_2 _values in the ICU population compared to the patients described in the EGDT study and other ED studies. Other interventions such as sedation and analgesia, most often to facilitate endotracheal intubation and ventilation, might have been beneficial for the balance between oxygen delivery and oxygen demand. Also, the trend of changing ScvO_2 _and other physiological values influencing outcome [23,24] is not taken into account in our study. Of course, all these factors are important differences between ER populations and ICU populations, but are not predominating. We are aware that comparison between those populations is limited by the above-mentioned differences.

As a result of the study design, statements about cut-off S(c)vO_2 _values for outcome prediction [20,24] or impact on therapeutic intervention are not possible. Also, we did not investigate the use of vital signs as indicator of tissue oxygenation in comparison to mixed or central venous saturation. Lack of clear insight of treatment and time spent at the different EDs, operating theatres or wards is a limitation of our study as well. Nevertheless, since we also aimed at the usefulness of measuring ScvO_2 _or SvO_2 _on ICU admission, we think these factors are not pertinent to the results. For example, Bracht *et al*. [20] found no correlation between ScvO_2 _values and length of hospital stay before unplanned ICU admission.

Comparing our sepsis population with the ED population described in the important study by Rivers [8] is meant to be purely illustrative. Obviously, as we described, there are differences between ED and ICU populations in general. But there are also, depending on geographical setting, important differences between populations and health care systems. Therefore, we subscribe to the view of Ho *et al*. [25] that the syndrome described in the EGDT trial may be relatively uncommon depending on geographical setting and health care system. However, this does not undermine the importance of early identification of patients at high risk for cardiovascular collapse. For example, in our study of the 14 patients with a ScvO_2 _< 50%, the in-hospital mortality was 57%.

Finally, the in-hospital mortality in our study was 32.0% for the total population and 27.0% for the patients with severe sepsis or septic shock. Again this reflects recent findings by others: Ho *et al*. (30.2%) [25] and Shapiro *et al*. (26.9%) [26].

## Conclusion

In conclusion, the incidence of low ScvO_2 _values for acutely admitted critically ill patients is low in Dutch ICUs. This is especially true for patients with sepsis/septic shock.

## Key messages

The incidence of low ScvO_2 _values of acutely admitted critically ill patients is low in Dutch ICUs. This is especially true for patients with sepsis or septic shock.

In our setting, use of ScvO_2_-guided resuscitation may only be helpful in a small subset of sepsis.

Mean SvO_2 _values and mean ScvO_2 _values in acutely admitted critically ill patients, including patients with severe sepsis or septic shock, were normal in our ICUs.

## Abbreviations

AMC = Amsterdam Medical Center; APACHE = acute physiology, age and chronic health evaluation; CCO = continuous cardiac output; CI = confidence interval; CVC = central venous catheter; CVP = central venous pressure; DO_2 _= (systemic) oxygen delivery; ED = emergency department; EGDT = early goal directed therapy; GH = Gelre Hospital; Hct = hematocrit; ICU = intensive care unit; MAP = mean arterial pressure; MCL = Medical Center Leeuwarden; MODS = multiple organ dysfunction syndrome; PAC = pulmonary artery catheter; SAP = systolic arterial pressure; SAPS = simplified acute physiology score; ScvO_2 _= central venous oxygen saturation; SvO_2 _= mixed venous oxygen saturation; S(c)vO_2 _= mixed/central venous oxygen saturation; SOFA = sequential organ failure assessment; VO_2 _= (systemic) oxygen consumption.

## Competing interests

The authors declare that they have no competing interests.

## Authors' contributions

PB drafted the manuscript, participated in coordination, and performed statistical analysis. JH was responsible for acquisition of patient data in AMC. MS participated in the design of the study and helped to draft the manuscript. CB provided general support. PS participated in the design of the study, was responsible for acquisition of patient data. in GH and helped to draft the manuscript. MK conceived of the study and participated in its design and coordination and helped to draft the manuscript.
